# Continuing development of vaccines and monoclonal antibodies against Zika virus

**DOI:** 10.1038/s41541-024-00889-x

**Published:** 2024-05-24

**Authors:** Sara E. Woodson, Kaitlyn M. Morabito

**Affiliations:** Division of Microbiology and Infectious Diseases, National Institute of Allergy and Infectious Diseases, National Institutes of Health, Bethesda, MD USA

**Keywords:** Biotechnology, Immunology

## Abstract

The 2016 Zika virus (ZIKV) epidemic catalyzed a global effort to develop diagnostic tests, vaccines, and therapeutic treatments. However, the rapid waning epidemiology of ZIKV stalled many countermeasure development efforts. On January 31 and February 1, 2023, the National Institute of Allergy and Infectious Diseases (NIAID) hosted “Continuing Development of Vaccines and Monoclonal Antibodies Against Zika Virus,” a workshop of assembled experts from multiple fields and sectors to review the latest ZIKV research findings and develop recommendations for advancing vaccines and monoclonal antibodies. This report describes the workshop proceedings and summarizes the key challenges and major recommendations identified at the workshop. While the current incidence and testing for ZIKV are low globally, ZIKV has not disappeared, and future large-scale outbreaks are possible. Developing an effective vaccine and monoclonal antibody treatment is still a public health priority, especially for persons who can become pregnant and who live or travel in ZIKV-endemic regions.

## Introduction

The Zika virus (ZIKV) was brought to global attention in 2016 when scientists discovered that ZIKV infections were linked to microcephaly and Guillain-Barre syndrome (GBS)^1–3^. ZIKV infection during pregnancy was later appreciated to cause a range of other congenital malformations^4–6^. ZIKV infection is also linked to Guillain-Barré syndrome, neuropathy, and myelitis in both children and adults. When an epidemic of ZIKV disease emerged in Brazil and other countries in the Americas between April 2015 and November 2016, the World Health Organization (WHO) declared ZIKV infection a Public Health Emergency of International Concern. By 2018, ZIKV had been listed as a priority disease in WHO’s R&D Blueprint regarding preparedness for infectious disease outbreaks. The 2016 ZIKV epidemic catalyzed a global effort to develop vaccines and therapeutic treatments^7,8^. Despite the unprecedented speed of countermeasure development for ZIKV, the epidemic waned before efficacy could be evaluated in human clinical trials and there is currently no approved treatment or vaccine against ZIKV. Countermeasures that can prevent ZIKV infection and disease remain a public health priority, particularly for persons who can become pregnant and who live or travel in ZIKV-endemic regions.

On January 31 and February 1, 2023, the National Institute of Allergy and Infectious Diseases (NIAID) hosted the workshop “Continuing Development of Vaccines and Monoclonal Antibodies Against Zika Virus,” which assembled experts in epidemiology, immunology, virology, healthcare, industry, non-profit administration, and government with the goals of updating countermeasure developers on the latest scientific information, encouraging new collaborations between academic researchers and industry, and providing a regulatory perspective on the development of countermeasures for ZIKV. The workshop participants aimed to assess the current state of knowledge of Zika virus infection and immunity, the development status and landscape of vaccines and monoclonal antibodies, and the challenges for advancing vaccines and monoclonal antibodies (mAb) (Supplemental Table 1). Session 1: “Zika Epidemiology” reviewed what is currently known about ZIKV epidemiology globally and reported findings from human natural history studies. Session 2: “Zika Infection and Immunity” highlighted recent scientific findings about ZIKV infection, pathogenesis, and the immune responses that confer protection. Session 3: “Zika and Dengue Immune Interactions” examined the relationship between ZIKV and DENV infections and the implications of this cross-reactivity for vaccine research. Session 4: “Vaccines and Antibody-based Countermeasures” surveyed the latest ZIKV vaccine and mAb landscape and updated participants on the most advanced development efforts. Wrapping up the two-day event, session 5: “Pathways for Advanced Countermeasure Development” offered considerations for advancing clinical development of ZIKV vaccines and mAbs, including regulatory pathways.

ZIKV has a high probability to reemerge on a broad scale like many other arboviruses, and scientists, clinicians, and public health experts need to be prepared to address future significant outbreaks or epidemics. This report highlights key findings and discussion points during the two-day workshop and concludes with a description of proposed next steps for vaccine and mAb development (Box 1).

Box 1 Summary of key takeaways and recommendations **Key takeaways and recommendations from Zika Virus Epidemiology and Congenital Infection Session:**

• ZIKV is still present in many regions and there is a need to restore diagnostic testing and surveillance globally. The full burden of ZIKV is unknown, in part because robust testing is not being conducted at local or national levels.

• Countries should prioritize differential diagnostic testing and focus on tests that are suitable for use in low incidence situations (i.e., tests that give fewer false positives).

• Natural history study protocols should be tailored for smaller outbreaks and positioned for rapid launch as soon as new outbreaks appear. Master protocols should also be considered to maximize efficiencies.

• Data should be gathered on baseline rates of neurodevelopmental abnormalities to provide a more accurate portrait of Zika-related birth defects.

• Investigators should collaborate and pool data from human cohort studies. Especially in the absence of a widespread outbreak that brings high case numbers, partnerships will be key to advancing the epidemiological landscape. More cohort studies are needed before efficacy trials for vaccines or monoclonal antibodies can likely proceed.

• Development of specific and sensitive diagnostics and reliable testing algorithms should continue.

• The effect of an outbreak, exposure, or infection on women’s behavior and choices regarding sexual activity, fertility, family planning, and pregnancy should be evaluated.

• Factors affecting early pregnancy loss are complicated and should be studied in more detail, including both virus-specific, such as virus strain, and host-specific factors such as gestational age, comorbidities, pregnancy history, etc.

• Surveillance resources should be directed toward comprehensively understanding ZIKV incidence and prevalence in humans, animals, and vectors in the Americas, Asia, and Africa, given the low transmission levels and periodic, but limited outbreaks in certain regions.

• Cost-effective surveillance options, especially in low-and-middle-income countries (LMICs), should be developed. For example, incorporation of ZIKV surveillance into wastewater surveillance planning or efforts should be considered.

• Re-establishing or continuing surveillance programs for ZIKV may face challenges for continued investment without having prevention or treatment candidates that can be implemented once infection incidence has increased.

**Key takeaways and recommendations from Zika Virus Infection and Immunity and the DENV and ZIKV Immune Interactions Sessions:**

• Focusing solely on antibody responses is an incomplete portrait of ZIKV immunity and T cell responses warrant further study.

• The limited access to NHPs has a particular impact on ZIKV research. Research into vaccinating pregnant persons remains challenging in the absence of these available animal populations or alternative models.

• Consider expanding the exploration and use of humanized rodent models, which have shown promise for ZIKV research and could provide a path forward should NHP accessibility remain a significant challenge.

• A comprehensive approach, including both innate and adaptive immune responses, to studying the immune response in ZIKV, particularly in the absence of clearly defined surrogates or correlates of protection, will be required.

• A better understanding of flavivirus maturation, including examining the potential impact of changes to the E protein sequence on flavivirus maturation and antigen structure could inform vaccine development.

• Continue to address the complexity of potential ZIKV and DENV cross-reactivity using cohort studies and refined, well-characterized assays.

**Key takeaways and recommendations from Vaccines and Antibody-based Countermeasures Session:**

• Vaccine and monoclonal development has stalled due to both the waning ZIKV incidence and limited resources to continue the development of countermeasures.

• Additional research is needed to understand the complexity of interactions between ZIKV and DENV immunity to ensure safe deployment of ZIKV vaccine(s) in DENV endemic regions.

• The target population and label indication need to be defined for each product.

• Because the vaccines and mAbs in clinical development have not yet been approved by regulatory authorities and have limited quantities of manufactured product, they would not be available for rapid mobilization to large populations in a future ZIKV outbreak.

• A key logistical obstacle for further development of vaccines and mAbs include the lack of available NHPs for research.

• Existential concerns about enhanced disease, in the context of DENV, should not slow ZIKV vaccine development efforts. Research to better understand the potential risk(s) can be done in parallel with development efforts.

• Vaccine deployment strategies, such as deploying ZIKV vaccines in conjunction with DENV vaccines, or vaccinating DENV seropositive individuals, could mitigate potential risks of enhanced disease from subsequent DENV infections.

**Key takeaways and recommendations from Pathways for Advanced Countermeasure Development:**

• Identifying a surrogate endpoint for accelerated approval for a ZIKV vaccine is a significant challenge that will likely require data from nonclinical and clinical studies, human challenge models, and seroprevalence surveillance.

• Product developers should engage the FDA early and often to discuss the potential pathways and requirements for their individual products.

• The clinical endpoints for trials and label indication for the product needs to be carefully considered and may include prevention of infection, prevention of disease, and prevention of congenital infection or disease.

• Prevention of infection or disease in vaccinees does not necessarily mean ZIKV vaccines will prevent congenital abnormalities or infection. More research is needed to understand the parameters that influence congenital infection.

• Indications for prevention of congenital infection or disease are unlikely for initial licensure given the logistical challenges of demonstrating this endpoint in phase 3 efficacy studies and it is anticipated that post-licensure or phase 4 studies would likely be needed.

• Vaccinating or treating pregnant or lactating persons has logistical and cultural challenges. Even strategies aimed at protecting persons of reproductive age before they become pregnant need to consider the safety of administration during pregnancy given the high number of unplanned pregnancies annually.

• Product developers should consider conducting development and reproductive toxicology (DART) studies early in the regulatory process and enrolling pregnant persons in phase 3 trials to help address safety in pregnant populations.

• When preparing for the next epidemic, it would be ideal to have an IRB-approved phase 3 protocol with an adequate supply of investigational product ready for distribution at key locations. Assuming those elements are in place, this level of preparedness would mark a significant and hopeful step forward in ZIKV epidemic readiness and response.

## Current state of knowledge regarding Zika virus epidemiology and congenital infection

The goal of this session was to provide an update on current ZIKV epidemiology and key findings from natural history studies.

As of December 2021, autochthonous mosquito borne transmission of ZIKV had been detected in 89 countries and territories globally, with 61 countries and territories reporting evidence of *Aedes aegypti* (the mosquito thought to primarily transmit ZIKV) but no documented autochthonous ZIKV transmission^9^. Currently, diagnostic testing and surveillance for ZIKV is low globally and the true prevalence of disease remains unknown. The COVID-19 pandemic hampered ZIKV diagnostic testing and surveillance efforts by diverting resources and modifying individual healthcare seeking behaviors, neither of which have rebounded as the pandemic has waned. Pan American Health Organization (PAHO) continues to actively collate and report ZIKV disease cases (https://www3.paho.org/data/index.php/en/mnu-topics/zika.html), but outside of the Americas, little new data are being reported, which may indicate low transmission or a lack of testing. Sporadic cases have been reported in the European Union and the Western Pacific, and India has reported several small outbreaks since 2016, with most recently 150 cases in Utter Pradesh state in October 2021^9,10^. In Brazil, the real burden of ZIKV disease remains unknown and is likely underreported, despite strong surveillance and diagnostic testing infrastructure. Brazil continues to see human cases, including some acute ZIKV disease, suggesting that the virus continues to have low-level circulation^11^. However, the full picture of ZIKV’s maintenance and circulation in Brazil remains to be elucidated as non-human primate and mosquito surveillance has sometimes shown discordant results (i.e., evidence of Zika infection or maintenance in either non-human primates or mosquitos, but not both populations in the same time or space) and is largely incomplete in many states^12–14^. More research is needed to explain ZIKV’s maintenance and circulation across many geographical regions.

The continental United States has seen no new reports of mosquito-borne ZIKV transmission since 2018, and few cases have been reported among travelers in the last 3 years. In the US territories, an average of 35 cases annually have been reported over the last 3 years, with recent cases only probable, not confirmed^15^. In a rare move, in May 2021 the FDA removed its requirement for routine ZIKV screening of US blood donations, citing the global decline in ZIKV incidence.

ZIKV-related congenital impacts are also not sufficiently understood. The US Zika Pregnancy and Infant Registry (USZPIR) is a US national surveillance system that tracks pregnant people within the US who have laboratory confirmed ZIKV infections and collects information from medical records about these pregnant people and their infants. This information includes physical examinations, neurodevelopmental screenings, assessments and evaluations, neuroimaging, hearing screenings, audiological evaluations, and ophthalmology examination. Using data from the USZPIR, investigators found that 4.6% of the infants tracked between 1 December 2015 and 31 March 2018 had a Zika-associated birth defect (ZBD), with no difference in outcome based on maternal symptom status (asymptomatic versus symptomatic). Among pregnancies with confirmed ZIKV infection, 6.2% of infants had a ZBD. In areas with widespread local transmission of the virus, ZBDs were 9 times higher than in areas without local transmission^4–6^. A limitation of findings from the USZPIR is a lack of a control group of pregnant persons who were not exposed to ZIKV during pregnancy. There are multiple challenges to the successful surveillance of ZBD and congenital Zika syndrome (CZS): a high proportion of ZIKV infections are asymptomatic, making surveillance difficult; laboratory serological testing is complicated by cross-reactivity with other flaviviruses and a lack of correlation between IgM and PCR test results; and consistent case definitions of microcephaly and other congenital abnormalities are still needed. The addition of congenital ZIKV infection to the Centers for Disease Control and Prevention’s (CDC) Birth Defects Surveillance Toolkit may help position public health and healthcare workers for better detection of ZBD in the future. Several human cohort studies launched when the 2016 outbreak was already peaking and were forced to adapt their endpoints or proceed with lower numbers after the infection rate waned. For example, the ZIP (Zika in Infants and Pregnancy) Trial (NCT02856984), initiated in June 2016, planned to enroll 10,000 pregnant persons but only enrolled 6,461 owing to the declining infection rate, which not only affected the number of cases but also led to missing data attributed to dwindling participant interest in the study and corresponding loss to follow-up. ZIKAlliance that consisted of eight Latin American partner countries at 14 sites, conducted its study when the outbreak peak had just passed, enrolling 3852 pregnant subjects before cohort numbers and samples dropped off rapidly (as presented at the workshop)^16,17^.

Considering the limited ZIKV cases, data from multiple cohort studies may need to be pooled or shared to reach sufficient case numbers for endpoints which will require harmonized or comparable testing algorithms. In preparation for future outbreaks, universal or master protocols should be put into place where possible, including standard definitions for key outcomes and terms such as ‘exposed pregnancy’. A master protocol is a single protocol that is designed to address multiple questions and could be used to directly compare multiple interventions as was done during the Ebola outbreak^18^.

Decision-makers will need to remain blinded in cohort studies is needed to facilitate flexibility and adaptability without bias. Questions that are still to be addressed include which testing strategy or algorithm is most efficient to detect ZIKV infection reliably with high confidence, what the role of seroconversion should have in surveillance studies, and how best to determine a baseline frequency for adverse pregnancy outcomes. The field should continue to pursue these research questions and elements in order to be prepared for future outbreaks.

## Current state of knowledge regarding Zika virus infection and immunity and the potential interactions between dengue virus and ZIKV immunity

The goals of these sessions were to set the baseline of what is known about ZIKV infection and protective immune responses, correlates of protection, and what basic research gaps remain. These sessions also assessed what is known about the impact of ZIKV immunity on dengue virus (DENV) disease severity and discussed considerations for Zika vaccines and mAbs.

A genome-wide approach to studying flavivirus-host interactions using primary human cells and patient-derived viruses has allowed researchers to characterize and compare transcriptional and epigenetic changes in infected versus uninfected cells. By separating out these different cell populations, researchers found that ZIKV targets transcription to block macrophage responses^19^. In addition, ZIKV infection increases the expression of genes enriched for lipid metabolism-related functions. In particular, researchers identified sterol regulatory element-binding protein (SREBP)-activated transcription as a mechanism for promoting ZIKV infection that could be agreeable to therapeutic targeting^20^.

DH1017.IgM is a novel pentameric ZIKV-specific IgM that was isolated from a pregnant woman infected with ZIKV. DH1017.IgM targets envelope (E) dimer epitope and provides new insights into potential effective and safe therapeutic candidates. The increased potency of the IgM antibody is related to the polyvalency of the IgM molecule; IgM appears more protective than an IgG with the same specificity^21^.

A study of two ZIKV DNA vaccine candidates found that while both vaccines elicited similar levels of neutralizing antibodies, only one vaccine was completely protective against a viral challenge in animal studies. This demonstrated that different vaccines can elicit qualitative differences in the composition of antibody responses^22,23^. Analysis of sera from non-human primates (NHP) and humans found differences in the vaccines’ capacities to elicit antibodies that can neutralize the structurally mature form of the ZIKV virion^23^. This discovery further suggests that the capacity to neutralize mature virions could be critically important for vaccine-induced immunity. A more comprehensive understanding of virion structure and dynamics could make it possible to generate vaccine candidates that direct immune responses to epitopes available on the mature viral particle.

Recent lessons applied from DENV research also support the idea that in vitro neutralization does not always equal protection. Historically, most flavivirus vaccine research has relied substantially on the presence of neutralizing antibodies measured by in vitro assays, but recent DENV studies suggest that serotype-specific neutralization may correlate better with protection of a given DENV serotype^24–26^. This highlights that both the quality and the type of neutralizing antibodies appear to matter for protection against disease, which is an important lesson to apply to ZIKV. In discussion, participants noted that the adoption of international standards for neutralization assays would allow studies to be better compared—such standards were not available in 2016 but could potentially be generated before the next major outbreak.

While scientists have made progress in understanding humoral immunity to ZIKV, T cells also play important roles in protection. One study examined whether prior dengue virus immunity impacted the kinetics and viral epitopes targeted by T cells elicited by ZIKV infection^27–29^. Memory T cell responses brought out either by prior infection with DENV or by immunization with a tetravalent live-attenuated DENV vaccine formulation recognize ZIKV-derived peptides^27–29^. Assessing such T cell reactivity could shed light on beneficial versus poor or harmful immune responses.

While structural proteins are typically the primary focus for studying humoral immunity, NS1 is also a potential vaccine antigen with a low risk for antibody-dependent enhancement (ADE), with some studies showing its potential to prevent severe flavivirus disease^30^. However, NS1 has not yet been explored as a broad-spectrum therapeutic antibody target and is not yet sufficiently understood. Structure-based vaccine and therapy design for NS1 are areas ripe for continued research.

NHP studies have provided important proof of concept that antibodies and vaccines can protect against congenital ZIKV infection. Using a rhesus macaque model of congenital ZIKV infection and mimicking real-world infection conditions, a ZIKV DNA vaccine was able to protect against prolonged maternal viremia and fetal pathology^31^. In the same model, two monoclonal antibodies did not eliminate maternal viremia but did limit vertical transmission, protecting the fetus from neurological damage^32^. Similarly, human IgG treatment controlled Zika viremia in pregnant rhesus macaques^33^. Development of suitable animal models has been widely challenged by the lack of available NHP colonies for research.

Infection with a low-passage African lineage ZIKV during pregnancy in mice more significantly affects fetal outcomes compared to Asian lineage ZIKV. Comparisons across seven ZIKV strains showed African-based strains had higher transmissibility in mosquitos and higher lethality in young and adult mice, compared to their Asian counterparts^34^. This finding could signal the higher epidemic potential of African ZIKV strains, and it also suggests that outbreaks caused by them could go undetected longer. Related research showed that high-dose exposure to African-lineage ZIKV caused pregnancy loss in macaques rather than birth defects^35,36^. More research is needed on gestational ZIKV infection in Africa to help understand the implications of these findings. African-lineage ZIKV strains could also be used to develop more stringent and consistent animal models, with endpoints based in pregnancy loss rather than the array of congenital abnormalities seen in live births.

Finally, ZIKV and DENV cross-reactive immunity is complex and needs further study. Lessons from natural infection studies—in particular, cohort studies in Nicaragua and Brazil—have shown that the immune response against ZIKV affect immune responses and disease outcomes during subsequent infections with DENV, and vice versa^37,38^. DENV infection appears to protect against uncomplicated ZIKV disease, but depending on the incoming DENV serotype, a prior ZIKV infection appears capable of one of several outcomes: it can increase the risk of symptomatic DENV infection; it can enhance DENV disease severity; or it can potentially protect against DENV^39^. Cross-reactive antibodies were stable or rose after a primary dengue and ZIKV infection and waned gradually after a secondary infection^40^. These findings complicate existing understandings of the interplay between anti-DENV and anti-ZIKV antibodies and disease outcomes. More research is needed to understand the immunological mechanisms of protection and risk across both diseases and to determine whether the findings from human cohort studies apply more broadly.

Flaviviruses are structurally heterogeneous and dynamic, constituting complex immunogenic targets. Recent research has further revealed this complexity, raising questions and generating insights for the development of vaccines and therapeutic treatments. Since no clear surrogates or correlates of protection have been identified, examining both innate and adaptive immune responses to ZIKV infection and vaccination will be important. Defining immunodominant epitopes and the ways in which neutralizing antibodies bind these epitopes will provide insights for vaccine development. With multiple immunological components contributing to ZIKV protection and immunity, and with multiple antibody functions likely contributing to protection, additional research is needed to broaden and strengthen understanding of ZIKV structure, transmission, and antigenicity. Working together will enable the field to continue advancing vaccines and therapeutic treatments in preparation for future outbreaks.

## Current development landscape of vaccines and monoclonal antibodies

The goal of this session was to hear from product developers on the status of their countermeasures in development and their plans, if any, to advance them in the future.

When the ZIKV epidemic emerged in 2016, the global research community leaped into action, mobilizing to develop safe and effective vaccines and therapeutics. Scientists made significant progress within an expedited time frame, identifying over 45 vaccine candidates in 2016–2017, with more than 25 candidates moved to preclinical studies and 12 candidates advanced to Phase 1 clinical trials (including DNA, mRNA, inactivated, live-attenuated, and vectored vaccine platforms)^7,8,41,42^. Currently, four vaccine candidates have moved or are moving into phase 2 clinical trials. Updates on three of the most advanced vaccine candidates were provided during this session. Research into potently neutralizing human mAbs also advanced during the same period and shows strong promise for treating and potentially preventing ZIKV infection. Now with low ZIKV incidence globally, advancing these medical countermeasures beyond phase II will be challenging, particularly given the difficulty of launching large clinical trials in the absence of a sustained outbreak or reliable prevalence data^42,43^.

Moderna’s investigational ZIKV vaccine, mRNA-1893, has successfully completed a phase 1 trial^44^. The vaccine induces a strong neutralizing antibody response comparable to levels observed during the acute phase of a ZIKV infection, and a detectable neutralizing antibody response was maintained one year following vaccination^45^. A phase 2 clinical trial is moving forward (NCT04917861) with 809 participants from the United States and Puerto Rico. This study will evaluate two administrations of mRNA vaccines at day 1 and day 29 at two dose levels (30 µg or 100 µg) compared to a single 100 µg dose administration and placebo 1.

Takeda’s purified inactivated Zika vaccine PIZV (TAK-426), currently undergoing clinical development, successfully completed a phase 1 clinical trial evaluating the safety, tolerability, and immunogenicity of three doses of PIZV. TAK-426 was well tolerated and immunogenic in both flavivirus-naive and flavivirus-primed adults. The magnitude and the kinetics of the high-dose (10-µg) PIZV-induced nAbs were comparable with those observed in convalescent ZIKV patients. Based on the safety and immunogenicity profiles of all TAK-426 doses assessed, the 10 μg TAK-426 dose was selected for further clinical development^46,47^.

In preclinical studies, two ZIKV DNA vaccine candidates developed by NIAID’s Vaccine Research Center, VRC5283 and VRC5288, were found to be immunogenic in mice and non-human primates^22,23^. Phase 1 clinical trials (VRC 319 and VRC 320) demonstrated that candidate VRC5283 had superior immunogenicity, with greatest T cell responses seen four weeks after a needle-free administration of the vaccine^48^. A Phase 2/2b clinical trial (VRC 705) of the VRC5283 candidate vaccine was initiated to assess safety, immunogenicity, and efficacy in endemic populations, but ZIKV incidence waned and despite implementing an adaptive enrollment approach, not enough cases were captured to assess vaccine efficacy. Interim data and epidemiological reviews concluded that there was a low possibility of documenting additional ZIKV infections and the trial was stopped early. Overall, only 3 out of 2338 subjects had virologically confirmed ZIKV infection (PCR). Assessment of the immunogenicity results from this trial are ongoing.

The identification of a subset of antibodies targeting the conformational epitope spanning the E dimer (EDE antibodies) that can potently neutralize both DENV and ZIKV have opened the door for next-generation flavivirus vaccine approaches^49^. Comparing the ZIKV and DENV immunologic structures opens a pathway for epitope-focused vaccine design that could elicit potent cross-neutralizing antibodies sufficient to protect against both ZIKV and DENV and the potential to mitigate antibody-dependent enhancement (ADE)^50,51^.

Potently neutralizing mAbs show promise as a tool for potentially preventing and/or treating ZIKV infection and recent capability demonstrations have shown proof-of-concept that mAb products could be rapidly identified and developed in response to future outbreaks^52^. ZIKV-117 (Vanderbilt University) has been shown to be highly potent and effective in preclinical studies, preventing fetal infection and demise in mice, protecting from and treating viremia in macaques, and has activity against both the Asian and African lineages of ZIKV^53,54^. Based on ID Biologics’ in silico and experimental biophysical analyses, ZIKV-117’s predicted half-life is 60–90 days, making it a strong candidate for further development. Currently, ZIKV-117 is moving forward in chemistry, manufacturing, and controls (CMC) development, in conjunction with relevant elements from WHO’s preferred product characteristics.

Further development of candidate vaccine and mAb products remain a challenge. Waiting for a widespread ZIKV outbreak to evaluate efficacy of candidate products will mean that a product will likely not be available in time for those who need it most, even when moving at highest possible capacity or speed. Barriers include the logistics of rapid manufacture and supply, product availability, the time requirements of clinical trial applications, and the unresolved question of the appropriate clinical endpoint (prevention of viremia in persons of child-bearing age versus reduction in incidence of congenital ZIKV syndrome). In addition to logistical barriers due to the current low circulation of ZIKV, this session identified key immunological, epidemiological, and regulatory questions that need to be addressed to further develop vaccines and mAbs against ZIKV. While neutralizing antibodies are commonly assumed to be a surrogate of protection, the mechanisms of protective immunity and the role of T cells for flavivirus vaccines are not fully understood. Most workshop discussants agreed that a desirable goal of vaccination or mAb treatment is prevention of congenital infection, which is a relatively rare outcome based on current data and likely not practical to assess in traditional phase 3 efficacy trials. A common theme of discussion among product developers and meeting discussants was what the appropriate endpoint(s) for efficacy and label indication would be for a given candidate product: prevention of disease in adults, prevention of infection in adults, or prevention of congenital infection or abnormalities. Safety issues for medical countermeasures also pose challenges to countermeasure development including concerns about ADE, Guillain-Barre syndrome, and issues specific to pregnant and lactating persons. Additional question about deployment strategies were also raised, including whether routine vaccinations should be used only in outbreak settings and which target populations should be prioritized for immunization.

## Pathways for advanced countermeasure development

The goals of this session were to identify considerations for advancing ZIKV vaccine and mAb development and explore the regulatory pathways for countermeasure development.

The final session of the workshop outlined potential licensure and approval pathways, focusing on research gaps that need to be filled and identifying opportunities and barriers to successful medical countermeasure development. Safety considerations and monitoring of outcomes will be crucial to the success of a ZIKV vaccine or mAb in all intended populations, which may include individuals of child-bearing age and pregnant or lactating persons. The longstanding exclusion of pregnant and lactating persons from clinical trial participation has given rise to their widespread reluctance to be immunized. If the target population for a ZIKV vaccine or mAb becomes persons who can become pregnant but who are not yet pregnant, safety remains a pressing concern since a large number of all pregnancies are unintended. In the USA, approximately half of all pregnancies are unintended. Without adequate safety information about ZIKV vaccines and mAbs during pregnancy, even strong medical countermeasure candidates will struggle to advance and critical target population will potentially be excluded from most of the clinical discussion.

There are ongoing efforts to provide evidence-based information about medications, vaccines, infections, and other exposures during pregnancy including MotherToBaby, a US network that collaborates with the Vaccines in Medications in Pregnancy Surveillance System (VAMPSS) to identify the circumstances in which a drug or immunization could cause harm^55–58^. Experience with other vaccines utilized during pregnancy can be leveraged to identify considerations for ZIKV vaccines and mAbs.

Even if ZIKV countermeasures are recommended solely for persons of reproductive age who can become pregnant and not for those who are pregnant, inadvertent pregnancy exposures are likely to occur since many pregnancies are unplanned. Data on safety in pregnancy and during lactation is still needed. In the absence of substantial trial data in pregnancy or lactation, a priori planned mechanisms for capturing observational data in a timely and systematic manner are important and should be a priority for public health and scientific experts.

Vaccine safety considerations become even more challenging outside the United States, especially in lower-resource settings. Microcephaly has been shown to have a higher prevalence in these settings, though how much can be attributed to ZIKV infection is unclear. A recent Guatemala-based study found a high prevalence of microcephaly or reduced head circumference in infants and young children, where a major risk factor was the presence of ZIKV antibodies in infancy, suggesting the possibility of either congenital or post-natal infection with ZIKV^59^. Determining clinical and safety endpoints beyond symptomatic infection for Phase III trials will be important and may also be key to encouraging vaccine uptake and the use of mAbs for prophylaxis and/or treatment. Safety endpoints of special interest in low-resource settings and LMICs include acute febrile illness, flavivirus-like illness hospitalizations, microcephaly, and developmental delays^60^. Such endpoints, especially in pediatric populations, are complicated, usually rare, and long term and will likely mean that researchers may need to follow infants through the major developmental years to monitor for adverse effects. These logistical factors must be taken into consideration for medical countermeasures when planning phase III or post-licensure trials, whether in the USA or elsewhere.

In the absence of substantial ZIKV transmission, there is a role for a controlled human infection model (CHIM) for ZIKV, an idea originally discussed in 2016 and published as an initial report. At the peak of the 2016 ZIKV epidemic when incidence was high, experts struggled to justify administering volunteers with infectious ZIKV^61^. When the epidemic rapidly waned, WHO announced support of the proposed CHIM study, noting that human challenge models could play a role in the regulatory pathway toward vaccines and therapeutics^62^. Researchers at NIAID and John Hopkins University isolated two clinical viral isolates from patients who presented with an uncomplicated Zika infection and evaluated them in NHPs for suitability in the CHIM. A clinical trial (NCT05123222) to establish a ZIKV CHIM has now begun, with extensive risk mitigation in place through the enrollment of flavivirus-naïve female subjects between ages 18 and 40, through the provision of reliable and confirmed birth control, and through the monitoring of subjects on an in-patient basis (initially until day 14, subsequently lowered to 8 days). The ZIKV CHIM appears promising to date and may provide efficacy data to support licensure.

From a regulatory perspective, several considerations must be in place for a ZIKV vaccine to advance along a regulatory pathway toward approval or licensure. Vaccine approval will follow the same paradigm as with other preventive vaccines, though unique considerations may apply if development occurs during a Public Health Emergency of International Concern (PHEIC). Depending on the licensure or approval pathway pursued, demonstration of clinical benefit for a ZIKV vaccine could be shown in disease endpoint efficacy studies, in studies that show an effect on a surrogate marker reasonably likely to predict clinical benefit, or in animal studies. Multiple challenges remain to be addressed: conducting a large phase 3 trial is difficult if not impossible in the absence of a widespread outbreak, a surrogate marker has not yet been established for ZIKV, and animal rule approval is very unlikely as a pathway for ZIKV vaccines to advance. Possible regulatory pathways forward for access to a ZIKV vaccine include accelerated approval, IND utilizing Expanded Access Regulations, and Emergency Use Authorization. For Accelerated Approval, a defined surrogate endpoint must be identified. Possible studies could include passive transfer of antibodies in animal models, challenge studies in animal models, or sero-epidemiologic studies. Assay precision, validation, and performance would need to be demonstrated and confirmatory trial design would need to be in place. For Investigational New Drug Application (IND) utilizing Expanded Access Regulations, there is a requirement of informed consent and IRB approval for all persons receiving an investigational vaccine. Emergency Use Authorization is unlikely in the absence of another large-scale outbreak. Product developers are encouraged to communicate early and often with the FDA’s Center for Biologics Evaluation and Research (CBER), and regulatory mechanisms are available to permit access to investigational ZIKV vaccines in the case of an outbreak.

A similar regulatory process applies to the approval of monoclonal antibody through the FDA’s Center for Drug Evaluation and Research (CDER). Important considerations for ZIKV-specific mAbs include identification of the target population and determination of appropriate endpoints for that target population. If multiple target populations are identified, efficacy endpoints could differ significantly between them and decisions will need to be made regarding prophylactic versus therapeutic treatment indication. Special consideration must be paid to any drug trials involving pregnant individuals, fetuses, and/or neonates. The design of the clinical program must take into account the indication(s) being pursued. For instance, there is a different risk/benefit analysis with prophylaxis versus treatment indications for ZIKV mAbs. In addition, the question of how to measure efficacy within the pregnant population has not yet been adequately answered—nor do researchers yet know if there a role for antiviral treatment in the management of congenital Zika syndrome. Product developers should engage early and frequently in dialogue with the FDA to discuss possible paths forward, working on a development plan in the context of an evolving understanding of ZIKV mechanism and structure, finalizing a plan for product use and indication, and mapping out proposed studies or trials to support that indication.

## Looking ahead: next steps for vaccines and monoclonal antibodies against Zika virus

ZIKV presents unique challenges from many of the other pathogens with epidemic potential: it is found on multiple continents; it has multiple clinical syndromes and is associated with several outcomes; it is largely asymptomatic in adults but causes significant congenital morbidity; it cross-reacts with other flaviviruses; and it lacks a licensed or approved product to prevent or treat infection. Additionally, advanced vaccine and mAb development is made exceptionally difficult owing to the current low global ZIKV incidence. An ideal and effective ZIKV vaccine or mAb must be capable of protecting fetuses from abnormalities, but its administration must also avoid causing potentially adverse side effects in this vulnerable population. While the ultimate goal is to prevent congenital disease, this endpoint is impractical to assess in phase 3 clinical trials currently, and so a more realistic indication and surrogate endpoint, such as prevention of infection in adults, will likely be required for licensure. Post-licensure studies will likely be required to monitor safety and effectiveness in pregnant and lactating people. More research is imperative to pinpoint the precise nature of the relationship between ZIKV and DENV infections. But the complex nature of these interactions should not prevent the advancement of potentially beneficial ZIKV vaccines and/or mAbs.

Despite these challenges, there are opportunities to advance the development of ZIKV vaccines and mAbs so that we will be better prepared for the next resurgence of ZIKV. Multiple vaccine candidates have been evaluated in phase 2 clinical studies. A newly established ZIKV CHIM shows significant promise for demonstrating efficacy of vaccines and mAbs in the absence of sustained ZIKV incidence and may provide additional insights into ZIKV disease and correlates of protection. There is now a better understanding of the immune responses generated by vaccination and infection and the broad spectrum of disease symptomologies. While many of these areas need additional research, the field is significantly more advanced than when ZIKV initially garnered an international response in 2016. International engagement not only of scientists and regulators but also of public health experts, civil society, and communities invested in the thriving and health of their populations will be required to continue advancement. Collective action and cooperative engagement, with a willingness to hear and listen to those with different experiences and expertise, will be essential to preventing the devastating health impacts of a new ZIKV outbreak on tomorrow’s children.

### Supplementary information

Supplementary Material

## Data Availability

Data sharing is not applicable to this article as no datasets were generated or analyzed during the current study.
