# Supplementary material for: Continuing development of vaccines and monoclonal antibodies against Zika virus
Source: NPJ Vaccines. 2024 May 24;9:91. doi: 10.1038/s41541-024-00889-x (PMC11126562; doi:10.1038/s41541-024-00889-x)
Supplement: Supplementary file 1 — Supplementary Material [file 41541_2024_889_MOESM1_ESM.pdf]

**Supplementary Table 1.**

**Workshop Agenda including Session presentations and speakers**

| Day 1 – January 31st, 2023 | | |
|---|---|---|
| **Time (EST)** | **Topic** | **Speaker** |
| 8:30 am | Welcome, Logistics, Workshop Goals | **Sara Woodson**<br>NIAID, NIH |
| 8:40 am | Opening Remarks from<br>NIAID's Division of Microbiology and Infectious Diseases Director | **Emily Erbelding**<br>NIAID, NIH |
| **Session 1: Zika Virus Epidemiology** | | |
| 9:00 am | Update on Epidemiology | **J. Erin Staples**<br>DVBD, CDC |
| 9:20 am | Zika in Infants and Pregnancy (ZIP) Study | **Martha Nason**<br>NIAID, NIH |
| 9:40 am | Congenital Zika Virus Infection: Data from the Centers for Disease Control and Prevention Surveillance | **Kate Woodworth**<br>DBDID, CDC |
| 10:00 am | ZIKV Exposure Determination in a Pregnant Women Cohort Setting – Lessons Learned from the ZIKAlliance Cohort | **Thomas Jaenisch**<br>University of Colorado School of Public Health |
| 10:20 am | Moderated Panel Discussion | **Moderators:**<br>Matthew Collins, Emory<br>Sara Woodson, NIAID |
| **Session 2: Zika Virus Infection and Immunity** | | |
| 11:00 am | Mechanisms Underlying Dendritic Cell Response to Zika Virus Infection | **Sujan Shresta**<br>La Jolla Institute of Immunology |
| 11:20 am | Structural Studies of Zika Virus in Complex with Neutralizing Antibodies | **Richard Kuhn**<br>Purdue University |
| 11:40 am | Zika Virus Immunity and Correlates of Protection | **Theodore Pierson**<br>NIAID, NIH |
| 12:00 pm | Protective Antibody Responses to Flaviviruses- Lessons from Dengue | **Aravinda deSilva**<br>University of North Carolina School of Medicine |
| 1:20 pm | Zika-Specific T Cell Response | **Daniela Weiskopf**<br>La Jolla Institute of Immunology |
| 1:40 pm | Flavivirus NS1 as a Target for Protective Antibodies | **Daniel Watterson**<br>University of Queensland |

| | | |
|---|---|---|
| 2:00 pm | Frequent Fetal Demise in Macaques Infected with African-Lineage ZIKV in the First Trimester | **David O'Connor**<br>University of Wisconsin - Madison |
| 2:20 pm | ZIKV DNA Vaccine and Monoclonal Antibodies Reduce Vertical Transmission and Improve Fetal Outcomes | **Koen Van Rompay**<br>University of California - Davis |
| 2:40 pm | Moderated Panel Discussion | **Moderators:**<br>Shelly Krebs, MHRP<br>Carlos Sariol, UPR<br>Laura VanBlargan, NIAID |
| **Session 3: Zika and Dengue Immune Interactions** | | |
| 3:20 pm | Immune Interactions Between Zika and Dengue: Insights from Natural Infection Studies | **Eva Harris**<br>University of California - Berkeley |
| 3:40 pm | Zika Virus Exposure Modulates Dengue Virus Immune Dynamics, Antibody Functionality, and Outbreak Risk | **Leah Katzelnick**<br>NIAID, NIH |
| 4:00 pm | Moderated Panel Discussion | **Moderators:**<br>Kim Dowd, NIAID<br>Kaitlyn Morabito, NIAID |
| 4:20 pm | Wrap up | **Kaitlyn Morabito**<br>NIAID, NIH |

| **Day 2 – February 1st, 2023** | | |
|---|---|---|
| **Time (EST)** | **Topic** | **Speaker** |
| 8:45 am | Welcome and Takeaways from Day 1 | **Kaitlyn Morabito**<br>NIAID, NIH |
| **Session 4: Vaccines and Antibody-Based Countermeasures** | | |
| 9:00 am | Zika Virus in Brazil: From Epidemiology to Interactions with Flavivirus | **Mauricio Nogueira**<br>Faculdade de Medicina de São José do Rio Preto |
| 9:30 am | Overview of the Zika Vaccine Landscape, Target Populations, and Target Product Profile | **Alan Barrett**<br>University of Texas Medical Branch |
| 10:00 am | Clinical Development of Moderna's Zika Virus Vaccine (mRNA-1893) | **Brett Leav**<br>Moderna Therapeutics |
| 10:20 am | Persistence of Immunogenicity of a Purified Inactivated Zika Virus Vaccine Candidate in Healthy Adults: 2 Years of Follow-up Compared with Natural Infection | **Camilo Acosta**<br>Takeda |
| 10:40 am | Development of Zika Virus DNA Vaccines | **Lesia Dropulic**<br>NIAID, VRC, NIH |

| | | |
|---|---|---|
| 11:20 am | Next Generation Vaccines:<br>Pan-Flavivirus or Non-Cross Reactive | **Gavin Screaton**<br>University of Oxford |
| 11:50 am | Human Monoclonal Antibodies for Prevention or Treatment of Zika Virus Infection | **James Crowe**<br>Vanderbilt University Medical Center |
| 12:10 pm | Moderated Panel Discussion | **Moderators:**<br>Gerry Kovacs, BARDA<br>Nancy Ulbrandt, NIAID |
| **Session 5: Pathways for Advanced Countermeasure Development** | | |
| 1:30 pm | Lessons Learned in Monitoring for Safety Outcomes Following Vaccination or Treatment for Viral Infections in Pregnancy and Lactation | **Christina Chambers**<br>University of California – San Diego |
| 1:50 pm | Clinical and Safety Endpoint Considerations for ZIKV Vaccine Phase III Trials in LMIC Countries | **Edwin Asturias**<br>University of Colorado School of Medicine |
| 2:10 pm | A Controlled Human Infection Model for ZIKV | **Stephen Whitehead**<br>NIAID, NIH |
| 2:50 pm | Clinical Development and Pathways for Licensure for Zika Virus Vaccines | **Kirk Prutzman**<br>CBER, FDA |
| 3:10 pm | Regulatory Considerations for the Development of Monoclonal Antibodies Against Zika Virus | **Natalie Pica,**<br>CDER, FDA |
| 3:30 pm | Moderated Panel Discussion | **Moderators:**<br>Anna Durbin, JHU<br>Kim Taylor, NIH |
| 4:00 pm | **Meeting Wrap-up** | **Sara Woodson,**<br>NIAID, NIH |
